# Health-related quality of life 15 years after oesophageal cancer surgery: a prospective nationwide cohort study

**DOI:** 10.1007/s11764-022-01257-1

**Published:** 2022-10-11

**Authors:** Anna SCHANDL, Zhao CHENG, Asif JOHAR, Pernilla LAGERGREN

**Affiliations:** 1 Surgical Care Science, Department of Molecular Medicine and Surgery, Karolinska Institutet, Retzius väg 13A, 4th floor, 171 77 Stockholm, Sweden; 2 Department of Anaesthesia and Intensive Care, 118 83 Stockholm Södersjukhuset, Sweden; 3 Department of Clinical Science and Education, 118 83 Stockholm Södersjukhuset, Karolinska Institutet Sweden; 4 Department of Surgery & Cancer, Imperial College London, London, UK

**Keywords:** EORTC QLQ-C30, EORTC QLQ-OES18, Functions, Long-term follow-up, Survivorship, Symptoms

## Abstract

**Purpose:**

We aimed to study oesophageal cancer survivors’ health-related quality of life (HRQL) 15 years after surgery and to identify factors related to reduced HRQL.

**Methods:**

A nationwide, prospective cohort study enrolling 616 patients who underwent open oesophageal cancer surgery in Sweden between April 2, 2001, and December 21, 2005. HRQL was evaluated by questionnaires 15 years after surgery. HRQL data for the 15-year survivors were individually matched for age, sex and comorbidity by using values from a Swedish background population. Multivariable linear regression models provided mean score differences (MSD) with 95% confidence intervals (CI) for each HRQL scale and item.

**Results:**

Among the 616 individuals in the original study group, 70 (11%) survived for 15 years and 52 (74%) responded to the questionnaires. Compared with a matched background population, the survivors reported problems in 10 of 25 HRQL aspects. Most of these were related to symptoms of the digestive tract, such as reflux (MSD 26.4, 95%CI: 18.3 to 34.4), dysphagia (MSD 17.7, 95%CI: 10.0 to 25.4) and eating difficulties (MSD 16.4, 95%CI: 11.3 to 21.4). Major postoperative complications after surgery were related to worse HRQL in 11 of 25 aspects.

**Conclusions:**

This study suggests that surgery for oesophageal cancer entails long-term, possibly life-long, symptoms related to the digestive tract.

Implication for Cancer Survivors.

Comprehensive support from healthcare may be imperative for oesophageal cancer survivors to adapt to and cope with consequences of oesophageal cancer surgery. Prevention, early identification and adequate treatment of postoperative complications may improve patient outcome.

## Introduction

Oesophageal cancer is the 7th most common cancer worldwide but is the 6th most lethal cancer type [1]. The 5-year survival is below 25%, mostly because of late symptom presentation and early metastatic spread [1, 2]. In high-income countries, adenocarcinoma represents approximately two-thirds of the oesophageal cancer cases, with high body weight, gastro-oesophageal reflux disease and Barrett’s oesophagus as key risk factors, while the remaining one-third consists of squamous cell carcinoma which is strongly linked with heavy smoking and alcohol consumption [1, 3]. The dominant curatively intended treatment involves surgical resection, often in combination with neoadjuvant chemotherapy or chemoradiotherapy [4]. The surgery is extensive and entails > 40% risk of postoperative complications [5, 6] and 5-year survival of 40% for patients without metastatic spread [1]. Postoperative recovery typically involves persistent symptoms and long-lasting detriments in health-related quality of life (HRQL) [7–10]. Previous studies have shown that 5 years after treatment, oesophageal cancer survivors suffer from symptoms such as eating difficulties, reflux, appetite loss and diarrhoea [11], problems which seem to persist up to 10 years after surgery [12]. So far, no prospective study has investigated oesophageal cancer survivors’ HRQL beyond that time point. Information on potential late- and long-term effects of cancer and its treatment has been stated to be one of the most important information needs among cancer survivors and their family members [13]. Further, knowledge about the clinical course of the disease is important for healthcare to provide adequate clinical counselling and to meet the long-term needs of the survivors. Therefore, we aimed to study oesophageal cancer survivors’ HRQL 15 years after surgery and to identify factors related to reduced HRQL.

## Methods

### Design

This nationwide prospective cohort study encompasses 90% of all oesophageal cancer patients in Sweden who underwent oesophagectomy between 1st April 2001 and 31st December 2005. At this time, no minimally invasive surgery was conducted, and all patients underwent open surgery of which most were operated on using transthoracic Ivor-Lewis oesophagectomy. Patients were followed up until death or the end of 2020. All patients who survived for 15 years after oesophageal cancer surgery were eligible for inclusion in the study. Written informed consent was obtained from all participants and the Regional Ethical Review Board in Stockholm, Sweden approved the study (Dnr 2015/0091–32). The Surgical Care Science patient research partnership group [14] provided comments from a patient perspective throughout the development of the publication.

### Data collection

A detailed description of this nationwide data collection can be found in other publications [15, 16]. In brief, the study was based on a complete, nationwide network of 174 Swedish hospital departments with contact clinicians involved in diagnostic procedures or treatment of patients with oesophageal cancer. Information regarding patient and tumour characteristics, treatment and complications were prospectively collected, and based on a predefined study protocol to ensure completeness and uniformity. Comorbidity was predefined as diabetes and cardiac, respiratory, renal or other specified conditions. Information about comorbidity was collected from the Swedish Patient Register [17] which contains all in-hospital diagnoses in Sweden since 1987 and all out-patient specialist care since 2001. The comorbidity diagnoses were verified by the patients at the 15-year follow-up. Data on postoperative complications were obtained through medical records and were defined as complications such as postoperative bleeding (exceeding 2 l or requiring reoperation), radiology or endoscopy verified anastomotic leakage, radiology-verified abscesses, sepsis, radiology-confirmed pneumonia, renal failure requiring dialysis, myocardial infarction confirmed with heart enzymes, radiology verified pulmonary embolism or stroke and respiratory failure requiring invasive ventilation, occurring within 30 days of surgery.

### Outcomes

HRQL was assessed 15 years after surgery using mailed, self-administered questionnaires, developed and validated by the European Organisation for Research and Treatment of Cancer Quality of Life (EORTC) [18, 19]. The EORTC Quality of Life Questionnaire-Core 30 (QLQ-C30) consists of 30 items for measuring HRQL aspects for cancer patients in general [18]. Questionnaire items are grouped into one global quality of life scale, five function scales (physical, role, emotional, cognitive and social), three symptom scales (fatigue, nausea/vomiting and pain) and six single items (dyspnoea, insomnia, appetite loss, constipation, diarrhoea and financial difficulties). An oesophageal cancer-specific questionnaire, the EORTC Quality of Life Questionnaire-Oesophageal 18 (QLQ-OES18) was used to assess problems specific for oesophageal cancer patients [19]. This 18-item questionnaire consists of four scales (dysphagia, reflux, eating difficulties and oesophageal pain) and six single items (trouble swallowing saliva, choking, dry mouth, coughing, speech difficulties and tasting problems). Both questionnaires had four response alternatives: ‘not at all,’ ‘a little,’ ‘quite a bit’ and ‘very much,’ except for the global quality of life scale, which has a seven-graded rating, ranging from 1 (‘very poor’) to 7 (‘excellent’). Questionnaire responses were linearly transformed into scores between 0 and 100, according to the scoring procedure in the EORTC manual [20]. In the global quality of life scale and the function scales, higher scores represent better HRQL, whereas higher scores in symptom scales and individual items correspond to more symptoms. Missing items were handled as recommended in the EORTC scoring manual [20].

Further, three study-specific questions were added to the questionnaire; *How well do you find yourself recovered after the surgery for oesophageal cancer?*, with the five response alternatives: ‘fully’; ‘almost’; ‘partly’; ‘not at all’ and ‘deteriorated’. This item was followed by the open-ended question; *If not, what problems remain*? and *How frequently do you recall that you have been treated for cancer?* with the options ‘daily’, ‘every week’, ‘every month’, ‘once a year’ and ‘never’.

### Background population

A random sample of Swedish adults (aged 40–79 years) was used to reflect the patient’s preoperative HRQL. The sample was drawn from the Swedish Population Register and was frequency-matched to reflect age and sex distribution of oesophageal cancer patients. The sample received the QLQ-C30, QLQ-OG25 and QLQ-OES18 questionnaires (overlapping questions and disease-related questions excluded) by mail. In total, 6969 individuals were eligible and 4910 responded to the questionnaires (70.5% participation rate) [21, 22]. For the current study, each oesophageal cancer survivor was individually matched, at the time of surgery, by age, sex and comorbidity (diabetes, cardiac, respiratory, renal or other specified conditions) to on average of 178 individuals (controls) from the background population (reference). Age matching was done at HRQL assessment, i.e. an individual who was 60 years old at the time of surgery, was matched to 75-year-old people at the 15-year follow-up. The matching of comorbidity was based on available patient registry data at follow-up.

### Statistical analysis

A senior biostatistician (A. Johar) with expertise in HRQL data management conducted all analyses. Multivariable linear regression models were used to calculate mean score differences (MSD) with 95% confidence intervals (CI). The analyses were adjusted for the potential confounders; age (at time of surgery), sex (men or women), comorbidity (0 or ≥ 1), tumour histology (squamous cell carcinoma/adenocarcinoma or dysplasia), tumour stage (0 to I, II to IV), surgical approach (transthoracic/transhiatal) and postoperative complications within 30 days of surgery (0 or ≥ 1). Evidence-based guidelines were used to determine the clinical relevance of the HRQL score deteriorations [23]. If no cut-off level was available, MSDs of 10 to 20 points were regarded as moderate or large clinical differences [24, 25]. To avoid multiple testing, statistical significance was tested at a 5% level of significance only if the MSDs were clinically relevant moderate or large. The study-specific questions were descriptively presented as numbers. All statistical analyses were conducted using SAS version 9.4 (Cary, NC).

## Results

### Patients

Of the 616 patients who were originally included in the cohort, 70 survived for at least 15 years. Among these, 52 (74%) responded to the HRQL questionnaires and were included in the present study. Characteristics of the survivors are presented in Table 1. As expected in a population of oesophageal cancer long-term survivors in Sweden, the majority were elderly (83%), of male sex (83%) and with adenocarcinoma or dysplasia (87%). The characteristics of the total cohort (616 patients) and the 15-year survivors were similar, apart from that patients in the total cohort had a higher tumour stage and age than the 15-year survivors (data not shown). Non-responders’ demographic characteristics were also similar to those with complete HRQL data.

**Table 1:** Characteristics of 15-year oesophageal cancer survivors undergoing surgical treatment in Sweden from 2001 to 2005

Characteristics	Categorisation	Oesophageal cancer survivors
		Number (%)
In total		52 (100)
Age at surgery	< 70 years	9 (17)
	≥ 70 years	43 (83)
Sex	Women	9 (17)
	Men	43 (83)
Comorbidity	0	29 (56)
	≥ 1	23 (44)
Tumour histology	Squamous cell carcinoma	7 (13)
	Adenocarcinoma or dysplasia	45 (87)
Tumour stage	0–I	29 (56)
	II-IV	23 (44)
Surgical approach	Transthoracic	43 (83)
	Transhiatal	9 (17)
Postoperative complications	0	35 (67)
	≥ 1	17 (33)

Comorbidity was categorised in no or 1 or more of the following conditions: diabetes, cardiac, respiratory, renal or other specified. Postoperative complications were defined as no or 1 or more of the following complications: postoperative bleeding (exceeding 2 l or requiring reoperation), radiology or endoscopy verified anastomotic leakage, radiology-verified abscesses, sepsis, radiology-confirmed pneumonia, renal failure requiring dialysis, myocardial infarction confirmed with heart enzymes, radiology verified pulmonary embolism or stroke and respiratory failure requiring invasive ventilation, occurring within 30 days of surgery

### HRQL 15 years after oesophagectomy

Compared with a matched background population, the 15-year survivors reported largely more symptoms in 10 out of 25 HRQL aspects, most of which were related to the digestive tract, such as reflux (MSD 26.4, 95%CI: 18.3 to 34.4), dyspnoea (MSD 15.1, 95%CI: 6.0 to 24.4), appetite loss (MSD 14.6, 95%CI: 6.8 to 22.4), nausea/vomiting (MSD 12.5, 95%CI: 6.2 to 18.6) and diarrhoea (MSD 8.7, 95%CI: 2.8 to 14.6). The 15-year survivors also reported more moderately more symptoms of dysphagia (MSD 17.7, 95%CI: 10.0 to 25.4), eating difficulties (MSD 16.4, 95%CI: 11.3 to 21.4), oesophageal pain (MSD 13.1, 95%CI: 6.7 to 19.6), trouble swallowing saliva (MSD 14.4, 95%CI: 6.5 to 21.7) and dry mouth (MSD 18.2, 95%CI: 9.8 to 13.6) (Table 2).

**Table 2:** Comparisons in health-related quality of life (HRQL) between 15-year oesophageal cancer survivors and an age-, sex- and comorbidity-matched background population presented as mean score differences (MSD) with 95% confidence intervals (CI)

HRQL aspects	Background population	15-year cancer survivors	HRQL differences
	Mean scores with 95%CI	Mean scores with 95%CI	Adjusted MSDs with 95% CI
EORTC QLQ-C30			
Global quality of life	77.1 (74.8–69.2)	69.2 (63.0–75.5)	− 7.8 (− 14.3 to − 1.4)
*Functional scales*			
Physical function	85.2 (82.9–87.5)	77.3 (70.3–84.3)	− 7.9 (− 14.9 to − 0.8)
Role function	86.9 (84.7–89.2)	77.2 (68.4–86.0)	− 9.7 (− 18.2 to − 1.2)
Emotional function	88.7 (87.5–89.9)	77.2 (68.5–89.9)	− 3.8 (− 8.8 to 1.2)
Cognitive function	86.9 (85.8–88.0)	79.8 (73.6–86.1)	− 7.1 (− 13.3 to − 0.9)
Social function	91.4 (90.1–92.6)	80.8 (72.3–89.3)	− 10.6 (− 19.0 to − 2.2)
*Symptom scales*			
Fatigue	20.1 (17.8–22.3)	32.1 (24.4–39.7)	12.0 (4.0 to 20.0)
Nausea/vomiting	2.2 (1.8–2.6)	14.7 (8.3–21.2)	12.5 (6.2 to 18.9)^b^
Pain	19.0 (16.0–22.0)	16.3 (10.6–22.0)	− 2.7 (− 9.4 to 4.0)
*Symptom items*			
Dyspnoea	16.9 (13.7–20.2)	32.1 (23.2–40.9)	15.1 (6.0 to 24.4)^a^
Insomnia	16.1 (14.8–17.5)	26.9 (18.9–34.9)	10.8 (2.9 to 18.7)
Appetite loss	3.4 (2.7–4.0)	17.9 (10.3–25.6)	14.6 (6.8 to 22.4)^b^
Constipation	6.1 (5.4–6.7)	13.1 (5.3–20.8)	7.0 (− 0.6 to 14.7)
Diarrhoea	5.0 (4.2–5.8)	13.9 (8.0–19.4)	8.7 (2.8 to 14.6)^b^
Financial difficulties	2.1 (1.5–2.7)	10.9 (3.8–17.9)	8.8 (1.9 to 18.7)
EORTC QLQ − OES18			
*Disease* − *specific symptom scales*			
Dysphagia	0.7 (0.5–0.8)	18.4 (10.6–26.1)	17.7 (10.0 to 25.4)^b^
Reflux	6.5 (5.6–7.1)	32.7 (24.8–40.6)	26.4 (18.3 to 39.4)^a^
Eating difficulties	2.1 (1.8–2.3)	18.4 (13.4–23.4)	16.4 (11.3 to 21.4)^b^
Oesophageal pain	3.7 (3.1–4.3)	16.9 (10.6–23.2)	13.1 (6.7 to 19.6)^b^
*Disease-specific items*			
Trouble swallowing saliva	1.3 (0.9–1.7)	15.4 (7.7–23.1)	14.1 (6.5 to 21.7)^b^
Choking	4.4 (3.7–5.1)	12.1 (6.4–18.0)	7.8 (1.9 to 13.6)
Dry mouth	12.5 (10.9–14.2)	30.8 (22.4–39.2)	18.2 (9.8 to 26.6)^b^
Coughing	14.6 (12.8–16.5)	21.8 (14.0–29.6)	7.2 (− 0.8 to 15.1)
Speech difficulties	2.6 (2.1–3.1)	5.1 (0.9–9.4)	2.5 (− 1.7 to 6.7)
Taste problems	1.4 (1.0–1.8)	7.7 (1.4–14.0)	6.3 (0 to 12.6)

^a^Clinically relevant and statistically significant large differences

^b^Clinically relevant and statistically significant moderate differences[23]

### Factors related to long-term HRQL problems

**Fig. 1:**
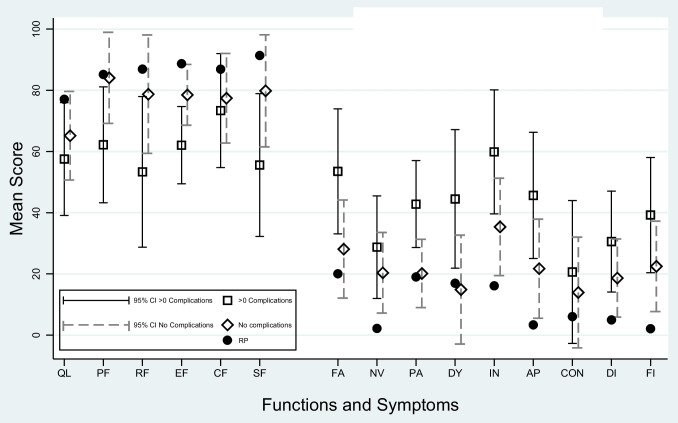
Adjusted results for global quality of life, functional scales and symptom scales and items 15 years after oesophageal cancer surgery categorised by postoperative complications, without postoperative complications and for the reference population (RP) presented as mean scores with 95% confidence intervals (CI). In global quality of life and functional scales, high scores indicate better HRQL. High scores in symptom scales and items correspond to more symptoms. QL = global quality of life; PF = physical function; RF = role function; EF = emotional function; CF = cognitive function; SF = social function; FA = fatigue; NV = nausea/vomiting; PA = pain; DY = dyspnoea; IN = insomnia; AP = appetite loss; CON = constipation; DI = diarrhoea; FI = financial difficulties

**Fig. 2:**
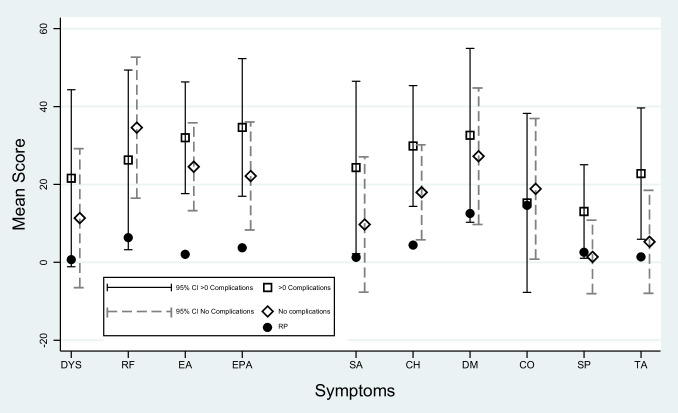
Adjusted results for oesophageal specific symptom scales and items in 15-year survivors after surgery categorised by postoperative complications, without postoperative complications and for the reference population (RP) presented as mean scores with 95% confidence intervals (CI). The higher scores, the more symptoms. DYS = dysphagia; RF = reflux; EA = eating difficulties; EPA = oesophageal pain; SA = trouble swallowing saliva; CH = choking; DM = dry mouth; CO = coughing; SP = speech problems; TA = taste problems

Major postoperative complications were related to worse HRQL in 11 out of 25 aspects (Tables 3, 4, 5). Those with postoperative complications entailed largely poorer social function (MSD − 24.2, 95%CI: − 43.2 to − 5.3), and moderately worse physical function (MSD − 21.9, 95%CI: − 37.3 to − 6.4), role function (MSD − 25.4, 95%CI: − 43.4 to − 5.3) and emotional function (MSD − 16.4, 95%CI: − 26.7 to − 6.1) when compared with those with no postoperative complications. There were large differences between the groups regarding fatigue (MSD 25.4, 95%CI: 8.8 to 42.0), pain (MSD 22.7, 95%CI: 11.1 to 34.2), dyspnoea (MSD 29.6, 95%CI: 11.2 to 48.0), appetite loss (MSD 23.9, 95%CI: 7.2 to 40.7) and moderate differences for financial difficulties (MSD 16.8, 95%CI: 1.4 to 32.1), speech difficulties (MSD 11.7, 95%CI: 1.9 to 21.4) and taste problems (MSD 17.5, 95%CI: 3.8 to 31.2), in favour of those without postoperative complications. The 15-year survivors with postoperative complications (*n* = 17) had the lowest HRQL scores, except for symptoms of reflux, which was more prevalent in those without complications. For the survivors without postoperative complications, physical function, pain, dyspnoea and speech difficulties were similar to those of the background population (Figs. 1, 2).

**Table 3:** Patient and clinical characteristics and health-related quality of life (HRQL) scores for *functions* in 15-year oesophageal cancer survivors presented as mean score differences (MSD) with 95% confidence intervals (CI)

EORTC QLQ-C30						
	Global quality of life	Physical function	Role function	Emotional function	Cognitive function	Social function
	MSD 95%CI	MSD 95%CI	MSD 95%CI	MSD 95%CI	MSD 95%CI	MSD 95%CI
Age						
≥ 70 years	Reference 1.0	Reference 1.0	Reference 1.0	Reference 1.0	Reference 1.0	Reference 1.0
< 70 years	− 5.3 (− 23.6 to 13.0)	2.0 (− 16.8 to 20.8)	− 10.0 (− 34.4 to 14.5)	− 18.2^b^ (− 30.8 to − 5.7)	− 2.0 (− 20.5 to 16.4)	− 9.3 (− 32.4 to 13.9)
Sex						
Men	Reference 1.0	Reference 1.0	Reference 1.0	Reference 1.0	Reference 1.0	Reference 1.0
Women	− 3.6 (− 21.8 to 14.6)	− 6.6 (− 25.3 to 12.1)	− 11.0 (− 35.2 to 13.4)	− 4.4 (− 16.9 to 8.0)	− 5.9 (− 24.3 to 12.5)	− 21.1 (− 44.1 to 1.9)
Comorbidity						
0	Reference 1.0	Reference 1.0	Reference 1.0	Reference 1.0	Reference 1.0	Reference 1.0
≥ 1	− 5.2 (− 18.7to 8.4)	− 1.0 (− 15.0 to 13.0)	0.8 (− 17.4 to − 19.0)	1.7 (− 7.6 to 11.0)	4.4 (− 9.4 to 18.1)	− 9.3 (− 26.5 to 7.9)
Tumour stage						
0–I	Reference 1.0	Reference 1.0	Reference 1.0	Reference 1.0	Reference 1.0	Reference 1.0
II–IV	6.1 (− 8.3 to 20.4)	− 0.6 (− 15.3 to 14.2)	1.0 (− 18.2 to 20.2)	1.2 (− 8.7 to 11.0)	0.1 (− 14.4 to 14.5)	− 0.2 (− 18.4 to 17.9)
Histology						
Squamous cell carcinoma	Reference 1.0	Reference 1.0	Reference 1.0	Reference 1.0	Reference 1.0	Reference 1.0
Adenocarcinoma	8.5 (− 11.8 to 28.7)	9.2 (− 11.7 to 30.0)	6.4 (− 20.7 to 33.5)	10.0 (− 3.9 to 23.9)	9.1 (− 11.4 to 29.6)	4.7 (− 21.0 to 30.3)
Surgical approach						
Transthoracic	Reference 1.0	Reference 1.0	Reference 1.0	Reference 1.0	Reference 1.0	Reference 1.0
Transhiatal	− 3.7 (− 21.8 to 14.4)	13.6 (− 5.1 to 32.2)	7.2 (− 17.0 to 31.4)	− 2.2 (− 14.6 to 10.3)	7.5 (− 10.8 to 25.8)	6.9 (− 16.0 to 29.8)
Postoperative complications						
0	Reference 1.0	Reference 1.0	Reference 1.0	Reference 1.0	Reference 1.0	Reference 1.0
≥ 1	− 7.6 (− 22.6 to 7.4)	− 21.9^b^ (− 37.3 to − 6.4)	− 25.4^b^ (− 45.4 to − 5.3)	− 16.4^b^ (− 26.7 to − 6.1)	− 4.0 (− 19.2 to 11.2)	− 24.2^a^ (− 43.2 to − 5.3)

Only clinically relevant differences were tested for statistical significance (*p* < 0.05)

^a^Clinically relevant and statistically significant *large* differences

^b^Clinically relevant and statistically significant *moderate* differences^23^

**Table 4:** Patient and clinical characteristics and health-related quality of life (HRQL) scores for *general symptoms* in 15-year oesophageal cancer survivors presented as mean score differences (MSD) with 95% confidence intervals (CI)

EORTC QLQ-C30									
	Fatigue	Pain	Nausea/vomiting	Dyspnoea	Insomnia	Appetite loss	Constipation	Diarrhoea	Financial difficulties
	MSD 95%CI	MSD 95%CI	MSD 95%CI	MSD 95%CI	MSD 95%CI	MSD 95%CI	MSD 95%CI	MSD 95%CI	MSD 95%CI
Age									
≥ 70 years	Reference 1.0	Reference 1.0	Reference 1.0	Reference 1.0	Reference 1.0	Reference 1.0	Reference 1.0	Reference 1.0	Reference 1.0
< 70 years	11.5 (− 8.7 to 31.8)	11.1 (− 3.0 to 25.1)	12.0 (− 4.7 to 28.6)	3.5 (− 19.0 to 25.9)	13.4 (− 6.7 to 33.5)	18.0 (− 2.4 to 38.5)	12.7 (− 10.2 to 35.7)	4.2 (− 11.9 to 20.4)	20.9^b^ (2.2 to 39.6)
Sex									
Men	Reference 1.0	Reference 1.0	Reference 1.0	Reference 1.0	Reference 1.0	Reference 1.0	Reference 1.0	Reference 1.0	Reference 1.0
Women	9.6 (− 10.6 to 29.7)	18.0^a^ (4 to 32.0)	22.8^a^ (6.3 to 39.3)	10.1 (− 12.2 to 32.5)	32.1^a^ (12.1 to 52.1)	6.9 (− 13.4 to 27.2)	1.6 (− 21.2 to 24.4)	6.7 (− 9.4 to 22.8)	17.7 (− 0.8 to 36.3)
Comorbidity									
0	Reference 1.0	Reference 1.0	Reference 1.0	Reference 1.0	Reference 1.0	Reference 1.0	Reference 1.0	Reference 1.0	Reference 1.0
≥ 1	2.5 (− 12.5 to 17.6)	4.3 (− 6.2 to 14.7)	13.6^b^ (1.2 to 26.0)	2.7 (− 14.0 to 19.4)	6.7 (− 8.2 to 21.7)	5.6 (− 9.6 to 20.8)	5.0 (− 12.4 to 22.5)	6.5 (− 5.8 to 18.8)	2.0 (− 11.9 to 15.9)
Tumour stage									
0–I	Reference 1.0	Reference 1.0	Reference 1.0	Reference 1.0	Reference 1.0	Reference 1.0	Reference 1.0	Reference 1.0	Reference 1.0
II–IV	− 3.7 (− 19.6 to 12.2)	7.2 (− 3.8 to 18.3)	9.3 (− 3.8 to 22.3)	− 0.8 (− 8.4 to 16.8)	0.9 (− 14.8 to 16.7)	− 2.6 (− 18.7 to 13.4)	− 1.0 (− 19.2 to 17.3)	1.8 (− 11.1 to 14.6)	3.7 (− 11.0 to 18.3)
Histology									
Squamous cell carcinoma	Reference 1.0	Reference 1.0	Reference 1.0	Reference 1.0	Reference 1.0	Reference 1.0	Reference 1.0	Reference 1.0	Reference 1.0
Adenocarcinoma	− 5.7 (− 28.1 to 16.8)	− 2.4 (− 18.0 to 13.3)	3.7 (− 14.8 to 22.1)	7.0 (− 17.9 to 31.9	− 6.3 (− 28.6 to 16.0)	− 16.7 (− 39.4 to 6.0)	1.2 (− 24.2 to 26.6)	− 9.0 (− 26.9 to 8.9)	− 13.5 (− 34.2 to 7.3)
Surgical approach									
Transthoracic	Reference 1.0	Reference 1.0	Reference 1.0	Reference 1.0	Reference 1.0	Reference 1.0	Reference 1.0	Reference 1.0	Reference 1.0
Transhiatal	− 13.0 (− 33.1 to 7.1)	2.1 (− 11.9 to 16.0)	− 4.3 (− 20.8 to 12.2)	− 28.1^a^ (-50.4 to − 5.9)	− 1.1 (− 21.0 to 18.8)	− 6.6 (− 26.9 to 13.7)	− 2.2 (− 24.9 to 20.6)	7.7 (− 8.4 to 23.7)	− 1.8 (− 20.4 to 16.7)
Postoperative complications									
0	Reference 1.0	Reference 1.0	Reference 1.0	Reference 1.0	Reference 1.0	Reference 1.0	Reference 1.0	Reference 1.0	Reference 1.0
≥ 1	25.4^a^ (8.8 to 42.0)	22.7^a^ (11.1 to 34.2)	8.4 (− 5.2 to 22.0)	29.6^a^ (11.2 to 48.0)	24.5^a^ (8.0 to 41.0)	23.9^a^ (7.2 to 40.7)	− 6.7 (− 25.8 to 12.4)	11.9 (− 1.6 to 25.4)	16.8^b^ (1.4 to 32.1)

Only clinically relevant differences were tested for statistical significance (*p* < 0.05)

^a^Clinically relevant and statistically significant *large* differences

^b^Clinically relevant and statistically significant *moderate* differences^23^

**Table 5:** Patient and clinical characteristics and health-related quality of life (HRQL) scores for *oesophageal cancer-specific symptoms* in 15-year oesophageal cancer survivors presented as mean score differences (MSD) with 95% confidence intervals (CI)

EORTC QLQ-OES18										
	Dysphagia	Reflux	Eating difficulties	Oesophageal pain	Trouble swallowing saliva	Choking	Dry mouth	Coughing	Speech difficulties	Taste problems
	MSD 95%CI	MSD 95%CI	MSD 95%CI	MSD 95%CI	MSD 95%CI	MSD 95%CI	MSD 95%CI	MSD 95%CI	MSD 95%CI	MSD 95%CI
Age										
≥ 70 years	Reference 1.0	Reference 1.0	Reference 1.0	Reference 1.0	Reference 1.0	Reference 1.0	Reference 1.0	Reference 1.0	Reference 1.0	Reference 1.0
< 70 years	7.1 (− 15.4 to 29.7)	− 9.3 (− 32.1 to 13.6)	4.3 (− 9.9 to 18.5)	8.3 (− 9.2 to 25.8)	− 13.3 (− 35.3 to 8.7)	− 2.5 (− 17.8 to 12.9)	− 15.5 (− 37.7 to 6.6)	3.0 (− 19.8 to 25.8)	− 4.0 (− 15.9 to 7.9)	10.2 (− 6.5 to 27.0)
Sex										
Men	Reference 1.0	Reference 1.0	Reference 1.0	Reference 1.0	Reference 1.0	Reference 1.0	Reference 1.0	Reference 1.0	Reference 1.0	Reference 1.0
Women	− 2.5 (− 24.9 to 20.0)	18.5 (− 4.3 to 41.2)	13.7 (− 0.4 to 27.9)	19.6^b^ (2.2 to 37.0)	5.1 (− 16.7 to 26.9)	18.7^b^ (3.4 to 34.0)	33.4^a^ (11.4 to 55.4)	− 6.5 (− 29.2 to 16.1)	1.9 (− 9.9 to 13.7)	5.3 (− 11.3 to 22.0)
Comorbidity										
0	Reference 1.0	Reference 1.0	Reference 1.0	Reference 1.0	Reference 1.0	Reference 1.0	Reference 1.0	Reference 1.0	Reference 1.0	Reference 1.0
≥ 1	− 0.4 (− 17.1 to 16.4)	4.7 (− 12.3 to 21.7)	5.5 (− 5.5 to 16.1)	2.3 (− 10.7 to 15.3)	5.2 (− 11.2 to 21.5)	− 5.3 (− 16.7 to 6.2)	14.8 (− 1.7 to 31.2)	1.8 (− 15.1 to 18.8)	0.5 (− 8.3 to 9.4)	− 5.3 (− 22.0 to 11.3)
Tumour stage										
0–I	Reference 1.0	Reference 1.0	Reference 1.0	Reference 1.0	Reference 1.0	Reference 1.0	Reference 1.0	Reference 1.0	Reference 1.0	Reference 1.0
II–IV	12.6 (− 5.1 to 30.3)	− 3.3 (− 21.2 to 14.6)	− 0.1 (− 11.3 to 11.1)	0.3 (− 13.4 to 14.1)	12.0 (− 5.2 to 29.3)	10.4 (− 1.7 to 22.4)	− 1.2 (− 18.5 to 16.2)	− 2.6 (− 20.4 to 15.3)	7.1 (− 2.2 to 16.5)	− 7.7 (− 20.8 to 5.4)
Histology										
Squamous cell carcinoma	Reference 1.0	Reference 1.0	Reference 1.0	Reference 1.0	Reference 1.0	Reference 1.0	Reference 1.0	Reference 1.0	Reference 1.0	Reference 1.0
Adeno − carcinoma	13.1 (− 11.9 to 38.1)	3.9 (− 21.5 to 29.2)	− 9.0 (− 24.8 to 6.8)	− 4.5 (− 23.9 to 14.9)	0.5 (− 23.9 to 24.8)	− 6.2 (− 23.3 to 10.8)	6.7 (− 17.9 to 31.2)	− 5.8 (− 31.1 to 19.4)	− 2.4 (− 15.6 to 10.9)	− 5.5 (− 24.1 to 13.0)
Surgical approach										
Transthoracic	Reference 1.0	Reference 1.0	Reference 1.0	Reference 1.0	Reference 1.0	Reference 1.0	Reference 1.0	Reference 1.0	Reference 1.0	Reference 1.0
Transhiatal	− 3.6 (-26.0 to 18.8	− 6.0 (− 28.7 to 16.7)	− 1.0 (− 15.1 to 13.1)	− 4.0 (− 21.4 to 13.3)	4.8 (− 17.0 to 26.6)	3.6 (− 11.6 to 18.9)	− 13.0 (− 34.9 to 8.9)	− 14.8 (− 37.4 to 7.8)	− 1.4 (− 13.2 to 10.4)	− 10.6 (− 27.2 to 6.0)
Postoperative complications										
0	Reference 1.0	Reference 1.0	Reference 1.0	Reference 1.0	Reference 1.0	Reference 1.0	Reference 1.0	Reference 1.0	Reference 1.0	Reference 1.0
≥ 1	10.2 (− 8.3 to 28.7)	− 8.3 (− 27.1 to 10.5)	7.4 (− 4.2 to 19.1)	12.5 (− 1.9 to 26.8)	14.6 (− 3.4 to 32.6)	11.9 (− 0.7 to 24.5)	− 6.7 (− 25.8 to 12.4)	5.4 (− 12.8 to 23.5)	11.7^b^ (1.9 to 21.4)	17.5^b^ (3.8 to 31.2)

Only clinically relevant differences were tested for statistical significance (*p* < 0.05)

^a^Clinically relevant and statistically significant *large* differences

^b^Clinically relevant and statistically significant *moderate* differences^24, 25^

Moreover, women reported more symptoms of nausea/vomiting, insomnia, dry mouth (large differences), pain, oesophageal pain and choking (medium differences), than men. The transhiatal approach of surgery was related to more symptoms of dyspnoea.

### The 15-year survivors’ perspectives of their recovery

Among the 50 survivors who responded to the study-specific questions, 17 stated that they had fully recovered from the cancer and its treatments, while 24 were almost recovered and 9 were partly recovered or had not recovered at all. Persistent eating problems or burdensome reflux were the most commonly stated reasons for not having obtained full recovery. More than half of the survivors (*n* = 27) did not recall the disease for more than once a year, and when they did, it was mostly related to positive feelings, such as being happy to have survived.

## Discussion

This study suggests that surgery for oesophageal cancer entails long-term, possibly life-long, symptoms associated with the digestive tract. Major postoperative complications were the single most important factor related to HRQL problems 15 years after surgery. Despite long-term symptoms and functional impairments, most patients stated that they were happy to have survived.

Despite the small number of 15-year survivors, the prospective, nationwide and population-based design with a relatively high participation rate (74%) counteracts selection bias, enables clinically meaningful conclusions and improves generalisability. The use of well-validated questionnaires reduces measurement bias. A potential study limitation is the lack of baseline HRQL data. However, preoperatively, patients may experience disease symptoms and may be emotionally influenced by the cancer diagnosis and ongoing neoadjuvant therapy. Therefore, obtaining HRQL data at that time point to mimic their normal wellbeing may be misleading. Instead, we used HRQL values from a background population to represent HRQL levels corresponding to what could be expected before the cancer surgery. To reduce the risk of more recent comorbidities influencing the results, we matched for comorbidity at time of follow-up. However, this choice may have induced a risk of over-adjustment because of cancer-related comorbidities. One might argue that some of the assessed symptoms are attributed to ageing. Therefore, the survivors were matched with individuals 15 years older, and this potential effect was also reduced by adjustments for age at the time of follow-up in the analyses. Smoking habits and socioeconomic status may differ between oesophageal cancer survivors and the background population. However, information on these potential confounders among the background population was not available and could therefore not be adjusted for. To reduce the risks associated with potential multiple testing of factors related to HRQL changes, we only tested for statistical significance if the changes were clinically relevant. However, there is a risk that the survivors’ perception of HRQL changes with time, by recalibration of personal standards and values, thereby, reconceptualizing their quality of life.

To the best of our knowledge, this is the first study that reports HRQL data for as long as 15 years after oesophageal cancer surgery. Because of the poor prognosis of the disease and that most patients are already elderly when they receive the cancer diagnosis, long-term follow-up studies are rare. One previous study evaluated mortality and HRQL 10 years after oesophageal cancer surgery between survivors with gastric tube reconstruction and whole stomach reconstruction in China. During the first years, gastric tube reconstruction was favourable, but in a longer-term perspective, HRQL were similar between the groups with remaining digestive tract symptoms [26]. Further, a Dutch study investigating long-term HRQL between patients who received surgery plus neoadjuvant chemo- and radio-therapy versus surgery alone with follow-up time exceeding 6 years, found function deteriorations and persistent symptoms independent of treatment regime [27]. Another previous study following this Swedish cohort 10 years after oesophagectomy showed reductions in most aspects of HRQL with gastro-intestinal symptoms as the most severe problems [12]. These symptoms may, at least to some extent, be explained by the permanent anatomical changes that the surgery entails. The loss of the gastric reservoir, the removal of the antireflux barrier of the gastric cardia, vagotomy and potential scarring of the proximal oesophagus may cause problems such as eating difficulties, reflux, dumping and dysphagia [28]. These permanent anatomical and physiological changes in combination with the long-lasting reported symptom burden indicates that patients who undergo oesophageal cancer surgery may expect to live with remaining symptoms for the rest of their lives. In order to adapt to the consequences of oesophageal cancer and its treatment which entail complex life changes, survivors and their family members may need comprehensive psychosocial support, such as counselling, education and group support [29].

In the present study, one factor related to worse HRQL was major postoperative complications following the surgery. It may seem somewhat surprising that HRQL would be influenced by complications that occurred 15 years ago. Yet, this finding is both confirmed and contradicted in the existing scientific literature. One recent European multicentre study including 362 patients, found that surgical complications were not associated with long-lasting symptoms following oesophageal cancer surgery [30]. On the other hand, one Swedish nationwide study including 92 oesophageal cancer survivors, suggested that postoperative complications were associated with considerable HRQL impairments up to 10 years after surgery [31]. Again, few long-term follow-up studies of oesophageal cancer patients have been published and in the existing studies, the sample size is small. Larger, preferably multicentre studies are warranted to be able to determine whether complications cause long-term reductions in HRQL. Oesophageal cancer resection is complex, and complications are common [6]. Minimal invasive surgery and centralization of the surgery are recommendations that may prevent complications [15, 32, 33]. However, early identification and adequate treatment of the complications are crucial to optimise the patient outcome [34]. Close postoperative surveillance and rapid management decisions taken by a multidisciplinary team of experts will benefit the patient and improve postoperative outcomes.

In conclusion, this study suggests that surgery for oesophageal cancer entails long-term persistent symptoms related to the digestive tract. Major postoperative complications were the most important factor for worse HRQL 15 years after surgery.

## Data Availability

No additional data are available.
